# Enhanced Upconversion Luminescence in Yb^3+^/Tm^3+^-Codoped Fluoride Active Core/Active Shell/Inert Shell Nanoparticles through Directed Energy Migration

**DOI:** 10.3390/nano4010055

**Published:** 2014-01-03

**Authors:** Hailong Qiu, Chunhui Yang, Wei Shao, Jossana Damasco, Xianliang Wang, Hans Ågren, Paras N. Prasad, Guanying Chen

**Affiliations:** 1 School of Chemical Engineering and Technology, Harbin Institute of Technology, Harbin 150001, China; E-Mails: qiuhailong2008@163.com (H.Q.); yangchh@hit.edu.cn (C.Y.); shoxxxa@gmail.com (W.S.); 2 Institute for Lasers, Photonics, and Biophotonics, University at Buffalo, State University of New York, Buffalo, NY 14260, USA; E-Mails: jossanad@buffalo.edu (J.D.); pnprasad@buffalo.edu (P.N.P.); 3 Department of Chemical and Biological Engineering, University at Buffalo, State University of New York, Buffalo, NY 14260, USA; E-Mail: xianlian@buffalo.edu; 4 Department of Theoretical Chemistry, Royal Institute of Technology, S-10691 Stockholm, Sweden; E-Mail: agren@theochem.kth.se

**Keywords:** upconversion, efficiency, core/shell, energy migration

## Abstract

The luminescence efficiency of lanthanide-doped upconversion nanoparticles is of particular importance for their embodiment in biophotonic and photonic applications. Here, we show that the upconversion luminescence of typically used NaYF_4_:Yb^3+^30%/Tm^3+^0.5% nanoparticles can be enhanced by ~240 times through a hierarchical active core/active shell/inert shell (NaYF_4_:Yb^3+^30%/Tm^3+^0.5%)/NaYbF_4_/NaYF_4_ design, which involves the use of directed energy migration in the second active shell layer. The resulting active core/active shell/inert shell nanoparticles are determined to be about 11 times brighter than that of well-investigated (NaYF_4_:Yb^3+^30%/Tm^3+^0.5%)/NaYF_4_ active core/inert shell nanoparticles when excited at ~980 nm. The strategy for enhanced upconversion in Yb^3+^/Tm^3+^-codoped NaYF_4_ nanoparticles through directed energy migration might have implications for other types of lanthanide-doped upconversion nanoparticles.

## 1. Introduction

Lanthanide-doped upconversion nanoparticles (UCNPs) are able to convert near infrared light (NIR) into shorter wavelength NIR, visible, and ultraviolet (UV) emissions, and have important technological applications ranging from bioimaging, displays, photovoltaics, drug delivery, biosensors, to photodynamic therapy [1,2,3,4,5]. Unlike multi-photon absorption, which requires high excitation density (~10^6^–10^8^ W/cm^2^) from ultrashort pulsed lasers, the upconversion (UC) processes generally involve the use of a much lower excitation density (~10^−1^–10^2^ W/cm^2^) (provided by a continuous-wave NIR diode laser) due to the use of the real ladder-like energy levels in lanthanides [1,4]. Typically, UCNPs are doped with ytterbium (Yb^3+^) sensitizer ions and activator ions such as erbium (Er^3+^), thulium (Tm^3+^), or holmium (Ho^3+^) [1,2,3,4,5]. The sensitizer ions are utilized to harvest NIR excitation and non-radiatively transfer their absorbed energy to the activator ions to produce luminescence. Despite recent progresses in accurate control of nanocrystal morphology and size [6,7], crystal phase [6], and emission colors [8,9,10], it remains a great challenge to achieve strong upconversion luminescence in UCNPs.

Attempts to overcome this challenge include variation of concentrations of sensitizer and activator dopants [11,12], selecting host lattice of low crystal phase and phonon energy [4], tailoring the local environment around lanthanide ions [13,14], or using noble metal nanostructures to enhance the energy transfer rate by surface plasmons [15]. However, none of these methods address surface-related quenching mechanisms that are pronounced in lanthanide-doped nanoparticles. Surface-related quenching mechanisms are associated with the fact that a majority of doped lanthanide ions are located on the nanoparticle surface due to the high surface/volume ratio created by the nanoscale dimension, and can be easily quenched by surface deactivations (such as surface impurities, ligands, and solvents). Coating nanoparticles with an inert shell is an effective strategy to minimize surface quenching due to the spatial isolation of the core nanoparticle from the surrounding environment [4]. Moreover, it has been demonstrated that the incorporation of sensitizer Yb^3+^ dopants in the inert NaGdF_4_ shell can further enhance the UC luminescence from the active core/inert shell (NaGdF_4_:20%Yb^3+^/2%Er^3+^)/NaGdF_4_ UCNPs [16]. This active core/active shell strategy has also been investigated in the BaGdF_5_:Yb^3+^/Er^3+^@BaGdF_5_:Yb^3+^ UCNPs [17], as well as in (NaYF_4_:Ce^3+^/Tb^3+^)/NaYF_4_:Ce^3+^ downconversion nanoparticles [18]. However, for UCNPs, the Yb^3+^ sensitizer in the active shell generally has to be lower than ~20% to avoid quenching effect produced by random excitation energy migration within the active shell to the surface deactivation sites on the outer surface of the active shell. The random excitation energy migration within the Yb^3+^ sub-lattice of the active shell is due to the unique two-energy-level structure of Yb^3+^ in association with its long-lived excited state [4]. The restriction of limited amount of Yb^3+^ sensitizer has impeded the way of increasing Yb^3+^ sensitizer in the active shell to multiply the efficiency of the active core/active shell UCNPs. Moreover, reports on UC luminescence increase using the active core/active shell strategy are limited to Yb^3+^/Er^3+^-doped nanoparticles, and there has been no report on other type of lanthanide-doped UCNPs.

Recently, fluoride nanoparticles doped with lanthanide ions have gained wide interests, as fluoride host lattice has high chemical and thermal stability and possesses low phonon cutoff energy, allowing the generation of relatively high UC radiations through curbing nonradiative losses at the intermediate states of lanthanide ions [6,7,8,9,10,11,12]. Among lanthanide-doped fluoride nanoparticles, Yb^3+^/Tm^3+^-doped NaYF_4_ nanoparticels are of particular interests, not only because NaYF_4_ has been shown to be the most efficient host material for UCNPs [7,19], but also because they display luminescence in the NIR, blue and UV range, which are useful for bioimaging [20], photoactivation [21], and light-activated therapy [22]. However, until this point, there has been no report on utilizing the active shell to increase the upconversion luminescence of Yb^3+^/Tm^3+^-doped NaYF_4_ UCNPs. In this work, we report on a new design of (NaYF_4_:Yb^3+^30%/Tm^3+^0.5%)/NaYbF_4_/NaYF_4_ active core/active shell/inert shell UCNPs. Unlike previously reported active core/active shell UCNPs with limited amount of Yb^3+^ concentration (20%) in the active shell, the active shell here incorporates 100% of Yb^3+^ ions to harvest excitation photon more efficiently. Moreover, as a result of the use of outmost inert NaYF_4_ shell to block the pathways to surface quenching cites, all energy migration within the NaYbF_4_ active shell layer have been directed to sensitize lanthanide ions in the core nanoparticles, yielding efficient UC luminescence about ~240 times higher than that of the typically used NaYF_4_:Yb^3+^30%/Tm^3+^0.5% UCNPs, and about 11 times brighter than that of the well-investigated active core/inert shell (NaYF_4_:Yb^3+^30%/Tm^3+^0.5%)/NaYF_4_ UCNPs.

## 2. Results and Discussion

### 2.1. Characterizations of Morphology and Crystal Structure

**Figure 1:**
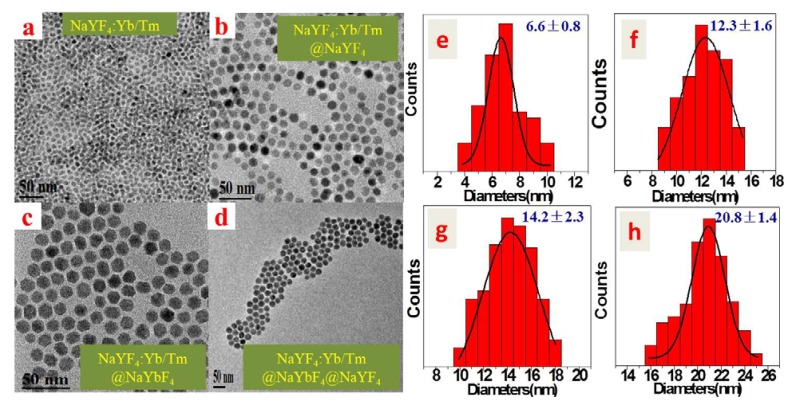
Transmission electron images (TEM) of: (**a**) The core NaYF_4_: 30%Yb^3+^, 0.5%Tm^3+^ nanoparticles; (**b**) The active core/inert shell (NaYF_4_: 30%Yb^3+^, 0.5%Tm^3+^)/NaYF_4_ nanoparticles; (**c**) The active core/active shell (NaYF_4_: 30%Yb^3+^, 0.5%Tm^3+^)/NaYbF_4_ nanoparticles; and (**d**) The active core/active shell/inert shell (NaYF_4_: 30%Yb^3+^, 0.5%Tm^3+^)/NaYbF_4_/NaYF_4_ nanoparticles. Histogram of size distribution of (**e**) The core NaYF_4_: 30%Yb^3+^, 0.5%Tm^3+^ nanoparticles; (**f**) The active core/inert shell (NaYF_4_: 30%Yb^3+^, 0.5%Tm^3+^)/NaYF_4_ nanoparticles; (**g**) The active core/active shell (NaYF_4_: 30%Yb^3+^, 0.5%Tm^3+^)/NaYbF_4_ nanoparticles; and (**h**) The active core/active shell/inert shell (NaYF_4_: 30%Yb^3+^, 0.5%Tm^3+^)/NaYbF_4_/NaYF_4_ nanoparticles. The size was evaluated according to TEM images of corresponding nanoparticles dispersed in hexane at a concentration of 0.1 wt.%.

Figure 1 shows the transmission electron microscopy (TEM) images of: (a) the core NaYF_4_: 30%Yb^3+^, 0.5%Tm^3+^ nanoparticles; (b) the active core/inert shell (NaYF_4_: 30%Yb^3+^, 0.5%Tm^3+^)/NaYF_4_ nanoparticles; (c) the active core/active shell (NaYF_4_: 30%Yb^3+^, 0.5%Tm^3+^)/NaYbF_4_ nanoparticles; and (d) the active core/active shell/inert shell (NaYF_4_: 30%Yb^3+^, 0.5%Tm^3+^)/NaYbF_4_/NaYF_4_ nanoparticles. As one can see, all four types of synthesized nanoparticles appear nearly spherical in shape and monodisperse. The average diameters of the core NaYF_4_: 30%Yb^3+^, 0.5%Tm^3+^, the active core/inert shell (NaYF_4_: 30%Yb^3+^, 0.5%Tm^3+^)/NaYF_4_, the active core/active shell (NaYF_4_: 30%Yb^3+^, 0.5%Tm^3+^)/NaYbF_4_, and the active core/active shell/inert shell (NaYF_4_: 30%Yb^3+^, 0.5%Tm^3+^)/NaYbF_4_/NaYF_4_ nanoparticles are determined to be about 6.6 ± 0.8, 12.3 ± 1.6, 14.2 ± 2.3, and 20.8 ± 1.4 nm, respectively. Compared to the size of the core NaYF_4_: 30%Yb^3+^, 0.5%Tm^3+^ nanoparticle, the size increase in the active core/inert shell and the active core/active shell nanoparticles indicates the successful growth of shell layer. In analogy, after coating an inert shell layer, the size of active core/active shell nanoparticles was increased from 14.2 to 20.8 nm, which suggests the formation of core/shell/shell structure. In addition, compared to the core of spherical shape, the core/shell (or core/shell/shell) nanoparticle has a similar spherical shape but at a larger size. Thus, it can be speculated that the shell layer in the core/shell (or core/shell/shell) nanoparticle is homogenous around the core nanoparticle.

**Figure 2:**
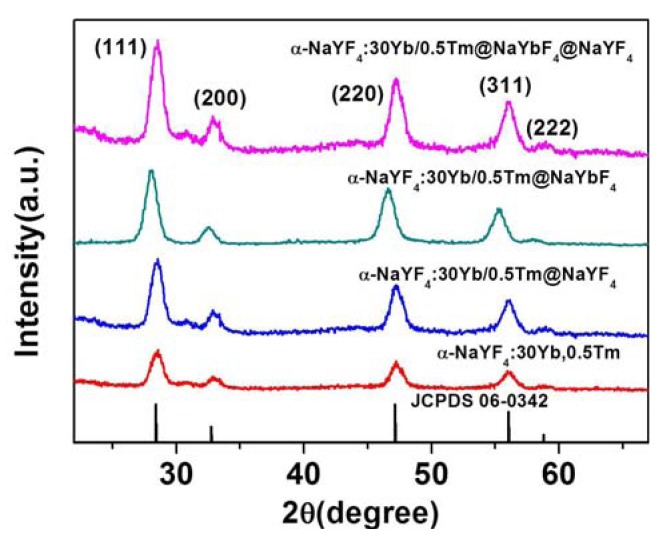
X-ray diffraction patterns of the core NaYF_4_: 30%Yb^3+^, 0.5%Tm^3+^ nanoparticles, the active core/inert shell (NaYF_4_: 30%Yb^3+^, 0.5%Tm^3+^)/NaYF_4_ nanoparticles, the active core/active shell (NaYF_4_: 30%Yb^3+^, 0.5%Tm^3+^)/NaYbF_4_ nanoparticles, and the active core/active shell/inert shell (NaYF_4_: 30%Yb^3+^, 0.5%Tm^3+^)/NaYbF_4_/NaYF_4_ nanoparticles, contrasted with the standard cubic NaYF_4_ structure of JCPDS 06-0342.

Figure 2 displays the X-ray diffraction (XRD) patterns of the core NaYF_4_: 30%Yb^3+^, 0.5%Tm^3+^ nanoparticles, the active core/inert shell (NaYF_4_: 30%Yb^3+^, 0.5%Tm^3+^)/NaYF_4_ nanoparticles, the active core/active shell (NaYF_4_: 30%Yb^3+^, 0.5%Tm^3+^)/NaYbF_4_ nanoparticles, the active core/active shell/inert shell (NaYF_4_: 30%Yb^3+^, 0.5%Tm^3+^)/NaYbF_4_/NaYF_4_ nanoparticles, as well as the standard JCPDS 6-0342 cubic structure of NaYF_4_. It is evident from the intensity of the peaks in Figure 2 that the prepared UCNPs are highly crystalline in nature. In addition, the positions of all XRD peaks correspond well to that of the standard JCPDS 6-0342 cubic pattern of NaYF_4_, and no peaks from other phases or impurities are observed.

### 2.2. Upconversion Luminescence

**Figure 3:**
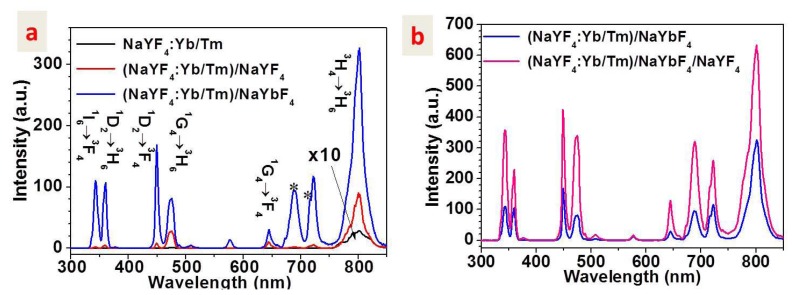
(**a**) Compared upconversion luminescence of colloidal nanoparticles (hexane dispersion, 1 wt.%) of the core NaYF_4_: 30%Yb^3+^, 0.5%Tm^3+^, the active core/inert shell (NaYF_4_: 30%Yb^3+^, 0.5%Tm^3+^)/NaYF_4_, and the active core/active shell (NaYF_4_: 30%Yb^3+^, 0.5%Tm^3+^)/NaYbF_4_. The peaks at ~690 and ~720 nm marked by asterisk correspond to the second order of the peaks at 345 and 360 nm; (**b**) Compared upconversion luminescence of colloidal nanoparticles (hexane dispersion, 1 wt.%) of the active core/active shell/inert shell (NaYF_4_: 30%Yb^3+^, 0.5%Tm^3+^)/NaYbF_4_/NaYF_4_ and the active core/active shell (NaYF_4_: 30%Yb^3+^, 0.5%Tm^3+^)/NaYbF_4_. The same batch of the core nanoparticles is utilized to grow the active core/inert shell, the active core/active shell, and the active core/active shell/inert shell nanoparticles. The excitation is performed with a diode laser at ~980 nm of about 50 W/cm^2^. All the spectra have been calibrated by the spectral sensitivity of the utilized spectrophotometer system.

**Figure 4:**
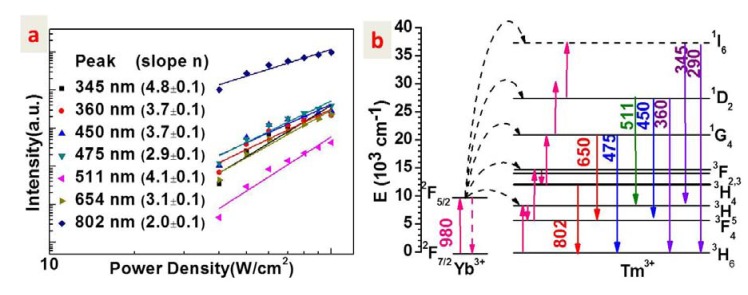
(**a**) A log-log plot of the dependence of various luminescence intensities from the active core/active shell/inert shell (NaYF_4_: 30%Yb^3+^, 0.5%Tm^3+^)/NaYbF_4_/NaYF_4_ nanoparticles on the excitation density; (**b**) Energy level diagrams of Yb^3+^ and Tm^3+^ ions as well as the involved mechanisms for upconversion luminescence from different energy states of Tm^3+^ ions.

Although NaYF_4_ host lattice of hexagonal phase are more efficient than its cubic phase [11], lanthanide-doped cubic NaYF_4_ nanocrystals are selected in the work as the resulting particle can be more easily produced for the size range of 5–20 nm that are essential for a broad spectrum of bio-related applications [3,4]. The UC luminescence spectra (in the spectroscopic range of 300–850 nm) of colloidal nanoparticles of the core NaYF_4_: 30%Yb^3+^, 0.5%Tm^3+^, the active core/inert shell (NaYF_4_: 30%Yb^3+^, 0.5%Tm^3+^)/NaYF_4_, and the active core/active shell (NaYF_4_: 30%Yb^3+^, 0.5%Tm^3+^)/NaYbF_4_ (hexane dispersion) are shown in Figure 3a. Six apparent UC emission bands are resolved, centered at 345, 360, 450, 475, 650 and 800 nm, which correspond to the ^1^I_6_→^3^F_4_, ^1^D_2_→^3^H_6_, ^1^D_2_→^3^F_4_, ^1^G_4_→^3^H_6_, ^1^G_4_→^3^F_4_, and ^3^H_4_→^3^H_6_ transitions of Tm^3+^ ions, respectively [7,11]. The weak emission at 511 nm arises from the transition of ^1^D_2_→^3^H_5_ of Tm^3+^ ions. The dependence of the intensities of these emission bands on the excitation power density indicates that five-, four-, three-, and two-photon processes are involved to populate the ^1^I_6_, ^1^D_2_, ^1^G_4_, and ^3^H_4_ state, respectively (Consult Figure 4a). The peaks at ~690 and ~720 nm marked by asterisk correspond to the second order of the peaks at 345 and 360 nm, respectively. We would like to highlight that the same batch of core nanoparticles is utilized to grow the active core/inert shell, the active core/active shell, and the active core/active shell/inert shell nanoparticles, allowing an accurate quantification of the effect of varying shell on the luminescence intensity of the core nanoparticle. The luminescence intensity (integrated over all the measured spectroscopic range) of the active core/inert shell (NaYF_4_: 30%Yb^3+^, 0.5%Tm^3+^)/NaYF_4_ nanoparticles is found to be about 23 times higher than that of the core NaYF_4_: 30%Yb^3+^, 0.5%Tm^3+^ nanoparticles, consistent with previously published results [23]. Remarkably, the luminescence intensity of the active core/active shell (NaYF_4_: 30%Yb^3+^, 0.5%Tm^3+^)/NaYbF_4_ is about 121 times higher than that of the core NaYF_4_: 30%Yb^3+^, 0.5%Tm^3+^ nanoparticles, and about 5.3 times higher than that of the active core/inert shell (NaYF_4_: 30%Yb^3+^, 0.5%Tm^3+^)/NaYF_4_ nanoparticles. Moreover, compared to the luminescence of the active core/inert shell (NaYF_4_: 30%Yb^3+^, 0.5%Tm^3+^)/NaYF_4_ nanoparticles, the NIR emission at 800 nm from the ^3^H_4_ state, the blue/red emission at 475/650 nm from the ^1^G_4_ state, the UV/blue emission at 360/450 nm from the ^1^D_2_ state, and the UV emission at 345 nm from the ^1^I_6_ state, is enhanced by about 3.2, 3.5, 21, and 57 times for the active core/active shell (NaYF_4_: 30%Yb^3+^, 0.5%Tm^3+^)/NaYbF_4_ UCNPs, respectively. The much higher enhancement times for luminescence involving higher-order multiphoton processes indicate that Yb^3+^ ions in the active shell can expedite upconverting processes in the core nanoparticles due to efficient migration of excitation energy from the active shell to the core nanoparticles.

Figure 3b shows the luminescence comparison result of the active core/active shell/inert shell (NaYF_4_: 30%Yb^3+^, 0.5%Tm^3+^)/NaYbF_4_/NaYF_4_ UCNPs and the active core/active shell (NaYF_4_: 30%Yb^3+^, 0.5%Tm^3+^)/NaYbF_4_ UCNPs. Luminescence at 800, 476/650, 365/450, and 345 nm from the ^3^H_4_, ^1^G_4_, ^1^D_2_, and ^1^I_6_ state in the active core/active shell nanoparticles are all enhanced by about two times. This luminescence enhancement, apparently, is a direct influence of the inert shell on the energy migrations inside the active shell, as the outmost inert shell is spatially isolated from the core nanoparticles. Combining results in Figure 3a,b, it can be easily found that the luminescence of the active core/active shell/inert shell (NaYF_4_: 30%Yb^3+^, 0.5%Tm^3+^)/NaYbF_4_/NaYF_4_ UCNPs is about ~240 times higher than that of the typically used NaYF_4_: Yb^3+^30%/Tm^3+^0.5% UCNPs, and about 11 times higher than that of the active core/inert shell (NaYF_4_: Yb^3+^30%/Tm^3+^0.5%)/NaYF_4_ UCNPs. It is noted that the luminescence comparison in Figure 3 is based on the same weight concentration of UCNPs dispersed in hexane. Thus, different number of the core, the active core/inert shell, the active core/active shell, the active core/active shell/inert shell UCNPs is involved for the luminescence comparison. If compared based on the same number of nanoparticles, higher enhancement times can be anticipated.

### 2.3. Upconversion Mechanism

Figure 4a displays a log-log plot of the dependence of the intensities of various emissions on the excitation density for the active core/active shell/inert shell UCNPs. In general, the number of photons which are required to populate the upper emitting state under unsaturated condition can be obtained by the relation [19]:

*I*_L_ ∝ *P*^n^ (1)

where *I*_L_ is the luminescence intensity, *P* is the pump laser power, and *n* is the number of the laser photons required. Hence, the slope values in Figure 4a can provide the information on the number of photons absorbed for upconversion. Slope values of 4.8, 3.7, 3.7, 2.9, 4.1, 3.1, and 2.0 are observed for UC emissions peaked at 345, 360, 450, 475, 511, 654, and 800 nm, respectively. This result illustrates that five-, four-, three-, and two-photon processes are involved to generate the UC emission at 345 (from the ^1^I_6_ state), 360/450/511 (from the ^1^D_2_ state), 475/645 (from the ^1^G_4_ state), and 800 nm (from the ^3^H_4_ state), respectively. These observations agree well with previous results of Yb^3+^/Tm^3+^-codoped NaYF_4_ materials [24]. Moreover, similar slope values were observed for the active core/active shell (NaYF_4_: 30%Yb^3+^, 0.5%Tm^3+^)/NaYbF_4_, and the active core/inert shell (NaYF_4_: 30%Yb^3+^, 0.5%Tm^3+^)/NaYF_4_ UCNPs (data not shown). The similar results indicate similar UC mechanisms for UV, visible, and NIR luminescence generation. Figure 4b shows the energy level diagrams of Yb^3+^ and Tm^3+^ ions as well as the proposed mechanisms for different luminescence bands. The Yb^3+^ ions firstly absorbs laser photons at ~980 nm, and are excited from the ground state ^2^F_7/2_ to the ^2^F_5/2_ state. Then, the Yb^3+^ ions in their excited states successively transfer the absorbed energy to the Tm^3+^ ion, and promote it from the ground sate to the ^3^H_5_ (first step), ^3^F_2,3_ (second step), ^1^G_4_ (third step), and ^1^D_2_ (fourth step) states, respectively. Multi-phonons from the NaYF_4_ host lattice are involved to assist the energy transfer from Yb^3+^ to Tm^3+^ ions. Nonradiative relaxations from the ^3^F_2,3_ state can populate the ^3^H_4_ state, from which the NIR emission at 800 nm is produced. In addition, the UV emission at 345 nm can be generated by radiative relaxation from the ^1^I_6_ state to the first excited state of ^3^F_4_. The UC luminescence at 360, 450, and 511 nm arise from radiative relaxations from the ^1^D_2_ state to the ground ^3^H_6_, the first excited state ^3^F_4_, and the second excited state ^3^H_5_, of Tm^3+^ ions, respectively. The blue emission at 475 nm and the red emission at 645 nm stems from radiative relaxations from the ^1^G_4_ state to the ground ^3^H_6_ state, and the first excited state ^3^F_4_, of Tm^3+^ ions, respectively.

### 2.4. Decay of Upconversion Luminescence

**Figure 5:**
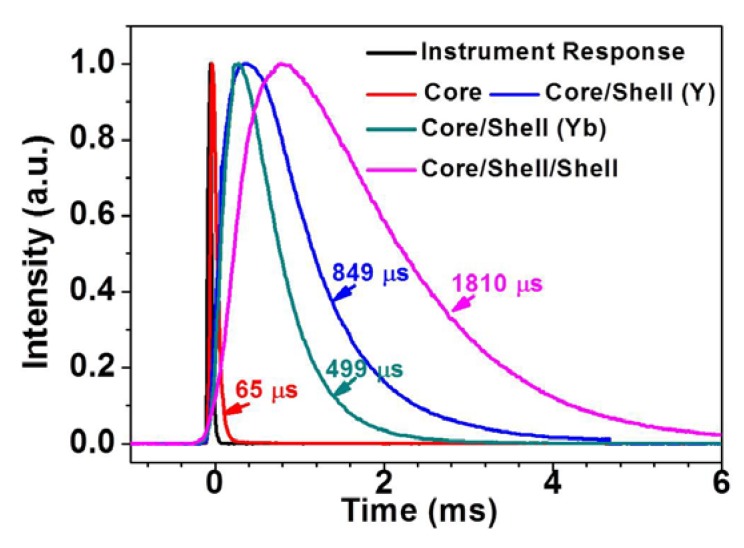
Decays of upconversion luminescence at 802 nm from the core NaYF_4_: 30%Yb^3+^, 0.5%Tm^3+^, the active core/inert shell (NaYF_4_: 30%Yb^3+^, 0.5%Tm^3+^)/NaYF_4_, the active core/active shell (NaYF_4_: 30%Yb^3+^, 0.5%Tm^3+^)/NaYbF_4_, and the active core/active shell/inert shell (NaYF_4_: 30%Yb^3+^, 0.5%Tm^3+^)/NaYbF_4_/NaYF_4_ nanoparticles.

Figure 5 displays the decays of upconversion luminescence at 802 nm from the core NaYF_4_: 30%Yb^3+^, 0.5%Tm^3+^, the active core/inert shell (NaYF_4_: 30%Yb^3+^, 0.5%Tm^3+^)/NaYF_4_, the active core/active shell (NaYF_4_: 30%Yb^3+^, 0.5%Tm^3+^)/NaYbF_4_ nanoparticles, and the active core/active shell/inert shell (NaYF_4_: 30%Yb^3+^, 0.5%Tm^3+^)/NaYbF_4_/NaYF_4_ nanoparticles.

**Figure 6:**
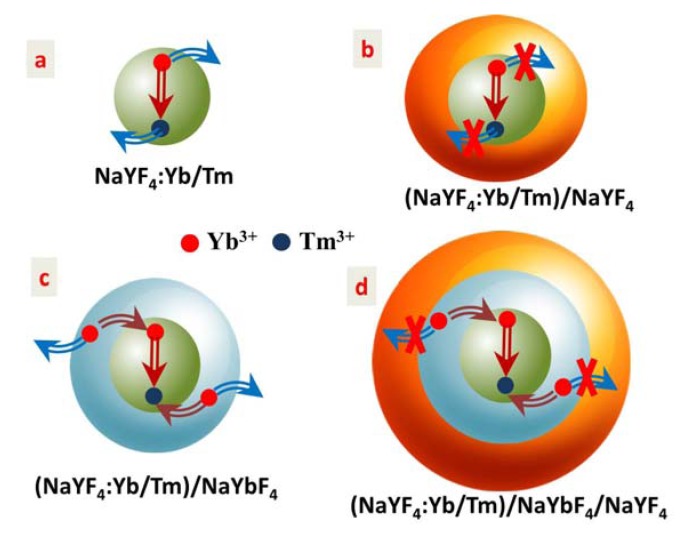
Schematic illustration of the luminescence quenching mechanism in: (**a**) The core NaYF_4_: 30%Yb^3+^, 0.5%Tm^3+^ nanoparticles, as well as the luminescence enhancement mechanism in (**b**) The active core/inert shell (NaYF_4_: 30%Yb^3+^, 0.5%Tm^3+^)/NaYF_4_ nanoparticles; (**c**) The active core/active shell (NaYF_4_: 30%Yb^3+^, 0.5%Tm^3+^)/NaYbF_4_ nanoparticles; and (**d**) The active core/active shell/inert shell (NaYF_4_: 30%Yb^3+^, 0.5%Tm^3+^)/NaYbF_4_/NaYF_4_ nanoparticles.

The average luminescence lifetime is determined to be about 65, 849, 499, 1810 μs, for the core, the active core/inert shell, the active core/active shell, and the active core/active shell/inert shell UCNPs, respectively. The significantly longer average lifetime of the active core/inert shell and the active core/active shell UCNPs than that of the core nanoparticles, demonstrate that surface-quenching effects in both type of core/shell structure have been significantly suppressed [25,26,27]. Note that despite having a thicker shell, the average lifetime of the active core/active shell nanoparticles is shorter than that of the active core/inert shell nanoparticles. This might be caused by the quenching effect produced by surface defects existed at the core surface. Due to a larger lattice mismatch between NaYbF_4_ and NaYF_4_ than between NaYF_4_ and NaYF_4_, a larger number of surface defects are anticipated at the core surface for the active core/active shell nanoparticle than for the active core/inert nanoparticle, thus leading to a shorter lifetime for the active core/active shell UCNPs. The growth of an inert shell on the active core/active shell nanoparticles can significantly increase the average lifetime of ^3^H_4_ state of Tm^3+^ located in the core nanoparticles. This might arises from a better protection of the core nanoparticles due to the larger spacing between the surface of the core and the surrounding environment. Moreover, due to the inert shell coating on the active core/active shell UCNPs, the excitation energy migration within the active shell is relatively confined (Consult Figure 6d), and, thus, become much stronger. The only way to dissipate the excitation energy in the active shell is to migrate it to the core nanoparticle surface (Consult Figure 6d). The stronger energy migration in the active core/active shell/inert shell nanoparticle results in the use of longer time to build up the population in the ^3^H_4_ state. This is in good agreement with the experimental observation in Figure 5 where a rising time of ~1 ms for the active core/active shell UCNPs is prolonged to be ~2.5 ms for the active core/active shell/inert shell UCNPs.

### 2.5. Quenching and Enhancing Mechanisms

Figure 6 illustrates the luminescence quenching mechanism in (a) the core NaYF_4_: 30%Yb^3+^, 0.5%Tm^3+^ nanoparticles, as well as the luminescence enhancement mechanism in (b) the active core/inert shell (NaYF_4_: 30%Yb^3+^, 0.5%Tm^3+^)/NaYF_4_ nanoparticles; (c) the active core/active shell (NaYF_4_: 30%Yb^3+^, 0.5%Tm^3+^)/NaYbF_4_ nanoparticles; and (d) the active core/active shell/inert shell (NaYF_4_: 30%Yb^3+^, 0.5%Tm^3+^)/NaYbF_4_/NaYF_4_ nanoparticles. For the core UCNPs without any shell coating (Figure 6a), numerous lanthanide dopants (sensitizer Yb^3+^ or activator Tm^3+^) are exposed to surface deactivations (caused by surface defects, lattice strains, as well as ligands and solvents that possess high phonon energy) owing to the high surface-to-volume ratio at nanometer dimension, thus yielding UC luminescence at low efficiency [4,26]. This surface-related quenching mechanism can be minimized by coating with an inert shell (Figure 6b), which can spatially isolate the core nanoparticles from the surface activations, yielding a higher UC efficiency. This is as the case of active core/inert shell (NaYF_4_: 30%Yb^3+^, 0.5%Tm^3+^)/NaYF_4_ nanoparticles here which is about 23 times more efficient than the core NaYF_4_: 30%Yb^3+^, 0.5%Tm^3+^ nanoparticles. In addition to minimization of surface-related quenching mechanism, in the active core/active shell nanoparticles, a large amount of sensitizer Yb^3+^ ions in the shell can further enhance the UC luminescence and boost population of high-lying energy levels through transferring its absorbed excitation energy to lanthanide dopants located in the core nanoparticles [16]. Indeed, the luminescence of the active core/active shell (NaYF_4_: 30%Yb^3+^, 0.5%Tm^3+^)/NaYbF_4_ is about 5.3 times higher in magnitude than that of the active core/inert shell (NaYF_4_: 30%Yb^3+^, 0.5%Tm^3+^)/NaYF_4_ nanoparticles, and exhibits much higher luminescence intensity ratio of the UV emission at 345 nm from the ^1^I_6_ state to the NIR emission at 800 nm from the ^3^H_4_ state (consult Figure 3a). However, the excitation energy harvested in the active shell can be dissipated by random energy migration in two ways; one way is to the core nanoparticle, and the other way is to surface deactivations at the outer active shell surface (Figure 6c). The way of random energy migration to the outer surface of the active shell can be cut off by further growing an inert shell (Figure 6d), directing energy migrations solely to the core nanoparticles and thus increasing the luminescence output, in good agreement with the experimental result in Figure 3b.

## 3. Experimental Section

### 3.1. Synthesis of NaYF_4_: Yb^3+^30%/Tm^3+^0.5% Core Nanoparticles

NaYF_4_ doped with 0.5%Tm^3+^ and 30%Yb^3+^ nanoparticles were synthesized using a modified co-thermolysis method [6]. All chemicals used in the synthesis were purchased from Sigma-Aldrich (Milwaukee, WI, USA) and used as received. One millimole amounts of Tm_2_O_3_, Yb_2_O_3_, and Y_2_O_3_ were mixed and dissolved in 10 mL 50% concentrated trifluoroacetic acid at 95 °C in a three-necked 100 mL flask. The molar compositions of the cationic lanthanide ions in the solution were [Y/(Tm + Yb + Y)] = 0.695, Tm/(Tm + Yb + Y)] = 0.005, and [Yb/(Tm + Yb + Y)] = 0.3. Then, the solution was evaporated to dryness under the argon gas purge. Next, 2 mmol CF_3_COONa, 6 mL oleic acid (90%, technical grade), 10 mL octadecene (90%, technical grade), and 6 mL oleylamine (70%, technical grade) were added into the three-necked flask. The resulting solution was then heated at 120 °C with magnetic stirring for 30 min to remove water and oxygen. The solution was then heated to 300 °C at a rate of about 12 °C per minute under argon gas protection, and kept at this temperature under vigorous stirring for about 0.5 h. A needle was used to let the argon gas out during the synthesis. The mixture was cooled to room temperature and precipitated by ethanol in an ultrasonic bath and collected by centrifugation at 7000 rpm for 5 min. The precipitate was then washed with ethanol for several times, and the nanoparticles were dispersed in 10 mL of hexane for further characterizations.

### 3.2. Synthesis of (NaYF_4_: Yb^3+^30%/Tm^3+^0.5%)/NaYbF_4_ Active Core/Active Shell Nanoparticles

The (NaYF_4_: Yb^3+^30%/Tm^3+^0.5%)/NaYbF_4_ active core/active shell nanoparticle were synthesized following a similar preparation procedure as the core. A 0.5 mmol amount of Yb_2_O_3_ dissolved in 10 mL 50% concentrated trifluoroacetic acid at 95 °C in a three-necked 100 mL flask. Then, the solution was evaporated to dryness under the argon gas purge. Next, 1 mmol CF_3_COONa, 0.5 mmol NaYF_4_: Yb^3+^30%/Tm^3+^0.5% core, 10 mL oleic acid, and 10 mL octadecene were added into the three-necked flask. The resulting solution was heated at heated at 120 °C with magnetic stirring for 30 min to remove water and oxygen. The solution was then heated to 300 °C at a rate of about 12 °C per minute under argon gas protection, and kept at this temperature, under vigorous stirring, for about 0.5 h. The mixture was cooled to room temperature and precipitated by ethanol in an ultrasonic bath and collected by centrifugation at 7000 rpm for 5 min. The precipitate was then washed with ethanol several times, and the nanocrystals were dispersed in 10 mL of hexane for further characterizations.

### 3.3. Synthesis of (NaYF_4_: Yb^3+^30%/Tm^3+^0.5%)/NaYF_4_ Active Core/Inert Shell Nanoparticles

The (NaYF_4_: Yb^3+^30%/Tm^3+^0.5%)/NaYF_4_ active core/inert shell nanoparticles were synthesized following a similar preparation procedure as described in Section 3.2. A 0.5 mmol amount of Y_2_O_3_ was dissolved in 10 mL 50% concentrated trifluoroacetic acid at 95 °C in a three-necked 100 mL flask. Then, the solutions were evaporated to dryness under the argon gas purge. Next, 1 mmol CF_3_COONa, 0.5 mmol NaYF_4_:Yb^3+^30%/Tm^3+^0.5% core, 10 mL oleic acid, and 10 mL octadecene were added into the three-necked flask. The resulting solution was then heated at heated at 120 °C with magnetic stirring for 30 min to remove water and oxygen. The solution was then heated to 300 °C at a rate of about 12 °C per minute under argon gas protection, and kept at this temperature under vigorous stirring for about 0.5 h. The mixture was cooled to room temperature and precipitated by acetone in an ultrasonic bath and collected by centrifugation at 7000 rpm for 5 min. The precipitate was then washed with ethanol for several times, and the nanocrystals were dispersed in 10 mL of hexane for further characterizations.

### 3.4. Synthesis of (NaYF_4_: Yb^3+^30%/Tm^3+^0.5%)/NaYbF_4_/NaYF_4_ Active Core/Active Shell/Inert Shell Nanoparticles

The (NaYF_4_: Yb^3+^30%/Tm^3+^0.5%)/NaYbF_4_/NaYF_4_ active core/active shell/inert shell nanoparticles were synthesized following a similar procedure as the core. A 0.25 mmol amount of Y_2_O_3_ dissolved in 50% concentrated trifluoroacetic acid at 95 °C in a three-necked 100 mL flask. Then, the solutions were evaporated to dryness under the argon gas purge. Next, 0.5 mmol CF_3_COONa, 0.25 mmol (NaYF_4_: Yb^3+^30%/Tm^3+^0.5%)/NaYbF_4_ active core/active shell nanoparticles, 5 mL oleic acid, and 5 mL octadecene were added into the three-necked flask. The resulting solution was then heated at heated at 120 °C with magnetic stirring for 30 min to remove water and oxygen. The solution was then heated to 300 °C at a rate of about 12 °C per minute under argon gas protection, and kept at this temperature under vigorous stirring for about 0.5 h. The mixture was cooled to room temperature and precipitated by ethanol in an ultrasonic bath and collected by centrifugation at 7000 rpm for 5 min. The precipitate was then washed with ethanol for several times, and the nanocrystals were dispersed in 10 mL of hexane for further characterizations.

### 3.5. Instruments

The size and morphology of UCNPs were characterized by transmission electron microscopy (TEM) using a JEM-2010 microscope (JEOL USA, Inc., Peabody, MA, USA) at an acceleration voltage of 200 KV. The powder X-ray diffraction (XRD) patterns were recorded by a Siemens D500 diffractometer using Cu Kα radiation (λ = 0.15418 nm). The 2θ angle of the XRD spectra was recorded at a scanning rate of 5°/min. UC luminescence spectra were recorded using a Fluorolog-3.11 Jobin Yvon spectrofluorometer (Horiba Jobin Yvon, Paris, France) with a slit width defining spectral resolution of 2 nm. The luminescence was excited at 975 nm using a fiber-coupled laser diode (Q-Photonics). All UC luminescence spectra have been corrected for the spectral sensitivity of the system. Decays of luminescence at 802 nm for UCNPs of the core NaYF_4_: Yb^3+^30%/Tm^3+^0.5%, the active core/active shell (NaYF_4_: Yb^3+^30%/Tm^3+^0.5%)/NaYbF_4_, the active core/inert shell (NaYF_4_: Yb^3+^30%/Tm^3+^0.5%)/NaYF_4_, the active core/active shell/inert shell (NaYF_4_: Yb^3+^30%/Tm^3+^0.5%)/NaYbF_4_/NaYF_4_ were recorded at the Infinium oscilloscope (Hewlett-Packard, Palo Alto, CA, USA) coupled to the photomultiplied tube (PMT) of the Fluorolog-3.11 Jobin Yvon spectrofluorimeter, using excitation at 975 nm from laser diode (Q-Photonics) operating in pulsed mode.

## 4. Conclusions

The intensity of UC luminescence of NaYF_4_: Yb^3+^30%/Tm^3+^0.5% UCNPs has been enhanced by ~240 times through utilization of the active core/active shell/inert shell (NaYF_4_: Yb^3+^30%/Tm^3+^0.5%)/NaYbF_4_/NaYF_4_ structure. Moreover, the UC luminescence of the active core/active shell/inert shell (NaYF_4_: Yb^3+^30%/Tm^3+^0.5%)/NaYbF_4_/NaYF_4_ UCNPs is determined to be about 11 times higher than that of well-investigated (NaYF_4_: Yb^3+^30%/Tm^3+^0.5%)/NaYF_4_ active core/inert shell UCNPs. Mechanistic investigation reveals that the significant UC enhancement in the active core/active shell/inert shell UCNPs arises from directed energy migration within the second active shell layer solely to the surface of the core nanoparticle. The guidance of energy migration is provided by the outmost inert shell which blocks the way of energy migration to surface quenching sites. The enhancement UC strategy demonstrated here in Yb^3+^/Tm^3+^-doped NaYF_4_ nanoparticles has implications to be extended to other type of lanthanide-doped UCNPs.
